# Immediate implant-retained prosthetic obturation after maxillectomy based on zygomatic implant placement by 3D-guided surgery: a cadaver study

**DOI:** 10.1186/s40729-021-00335-w

**Published:** 2021-06-14

**Authors:** N. Vosselman, H. H. Glas, S. A. H. J. de Visscher, J. Kraeima, B. J. Merema, H. Reintsema, G. M. Raghoebar, M. J. H. Witjes

**Affiliations:** Department of Oral and Maxillofacial Surgery, University of Groningen and University Medical Center Groningen, Hanzeplein 1, P.O. Box 30.001, 9700 Groningen, RB The Netherlands

**Keywords:** Maxillectomy, 3D VSP, Guided surgery, Zygomatic implants, Prosthetic rehabilitation, Maxillary obturator, Computer-aided design, Three-dimensional

## Abstract

**Background:**

The aim of this study was to introduce a complete 3D workflow for immediate implant retained prosthetic rehabilitation following maxillectomy in cancer surgery. The workflow consists of a 3D virtual surgical planning for tumor resection, zygomatic implant placement, and for an implant-retained prosthetic-obturator to fit the planned outcome situation for immediate loading.

**Materials and methods:**

In this study, 3D virtual surgical planning and resection of the maxilla, followed by guided placement of 10 zygomatic implants, using custom cutting and drill/placement-guides, was performed on 5 fresh frozen human cadavers. A preoperatively digitally designed and printed obturator prosthesis was placed and connected to the zygomatic implants. The accuracy of the implant positioning was obtained using 3D deviation analysis by merging the pre- and post-operative CT scan datasets.

**Results:**

The preoperatively designed and manufactured obturator prostheses matched accurately the per-operative implant positions. All five obturators could be placed and fixated for immediate loading. The mean prosthetic point deviation on the cadavers was 1.03 ± 0.85 mm; the mean entry point deviation was 1.20 ± 0.62 mm; and the 3D angle deviation was 2.97 ± 1.44°.

**Conclusions:**

It is possible to 3D plan and accurately execute the ablative surgery, placement of zygomatic implants, and immediate placement of an implant-retained obturator prosthesis with 3D virtual surgical planning.The next step is to apply the workflow in the operating room in patients planned for maxillectomy.

## Introduction

Surgical management and oral rehabilitation of patients diagnosed with a maxillary tumor is challenging. The size and extent of the maxillary defect, patient factors, and comorbidities are decisive factors for the choice of surgical, prosthodontic, or combined rehabilitation after a maxillectomy [1, 2]. Different treatment modalities have been described in the literature. Primary closure, prosthodontic rehabilitation by an obturator prothesis, or surgical rehabilitation by tissue grafting the maxillary defect, can be considered in order to obtain the best functional outcome for the patient [3].

While the functional results of prosthodontic and surgical rehabilitation of small- to medium-sized maxillary defects are somewhat comparable, reconstruction with free flaps seem to provide better speech and swallowing results for extensive or anterior located defects than conventional prosthetic obturation [4]. Supporting the obturator prostheses with implants improves the results of oral function rehabilitation significantly, as well as being a viable technique to improve the functionality of prosthetic rehabilitation in patients who have undergone a maxillectomy [5]. If conventional (obturator) prostheses are expected to be unsuccessful due to, e.g., lack of retention or load bearing problems of the (irradiated) soft tissues, implant placement to support the prosthesis should be pursued.

Unfortunately, implant survival in irradiated maxillary residual native bone seems less predictable in comparison to placement in the mandible (maxilla or mandible, 59% and 85%, respectively; *p* = 0.001) [6]. Remote anchorage in zygomatic bone appears to result in higher survival rates. The anchorage in higher level zygomatic bone, subject to lower or no radiation doses, seems favorable when the maxilla is exposed to post-operative radiotherapy [7, 8]. An advantage of the use of zygomatic implants after a maxillectomy is the possibility to obtain immediate prosthetic support. Many studies have reported that immediate prosthodontic rehabilitation after ablative surgery is of benefit for the patient [7–9]. Immediate loading of zygomatic implants is achievable due to a good primary stability in the bicortical plate of the zygoma complex [10, 11]. However, accurate freehand placement of zygomatic implants, taking the preferred prosthodontic positioning into account, is considered difficult. In the absence of guiding anatomic references, it is challenging to place two zygomatic implants at the defect side because of the long drill path.

With the availability of three-dimensional (3D) techniques, head and neck surgery-guided resections and implant placements, based on a preoperative virtual surgical plan (VSP), is becoming a standard of care [12]. 3D VSP enables a combination of ablation and reconstruction due to the knowledge of the pre-operative size of the defect. This permits 3D planned reconstructions with bone-containing free flaps and ideal prosthetic-driven placement of implants in one surgical procedure [12]. For ablative surgery combined with zygomatic implant placement and prosthetic rehabilitation, 3D virtual planning and computer-aided design have not yet been fully explored.

Although the production of 3D designed implant drill guides is now readily available, the shape and format of zygomatic implants demand a new guide design. The need for a prosthetic angulated implant head to obtain prosthetic support and an ideal screw emergence position on the palatal/occlusal surfaces, together with the implant length, as well as the need for multiple implants and, in oncological cases, the lack of supportive structures, make designing a guide challenging. To the best of our knowledge, a full workflow including tumor surgery, placing of zygomatic implants with 3D printed guides is currently not available. There have been no reports of the design, production, and application of personalized combined bone-supported drill and placement guides for zygomatic implants in order to transfer the virtual planning accurately.

The aim of this study was to develop a workflow that allows one procedure for tumor resection and rehabilitation by immediate placement of zygomatic implants in combination with an implant-retained surgical obturator, as a new treatment modality, using virtual surgical planning. This should lead to optimal placement for immediate loading of the obturator prosthesis as an end result. It is hypothesized that after maxillectomy, the introduction of custom drilling and placement guides for zygomatic implants provides accurate translation of a virtual planning allowing a one procedure workflow for immediate implant-retained prosthetic rehabilitation with a pre-planned obturator prosthesis.

## Materials and methods

Cone beam CT-datasets of the skulls of five fresh-frozen edentulous cadavers were obtained (Planmeca, ProMax 3D Max, Stockholm, Finland; 576 slices, voxel size 0.3 mm, FOV: 11 × 16 cm). The settings were in accordance with the clinical settings used for implant planning. A 3D model of the zygomatic bone and maxillae was created using ProPlan CMF 3.0 (Materialise, Leuven, Belgium) software.

### Virtual surgical planning

**Fig. 1:**
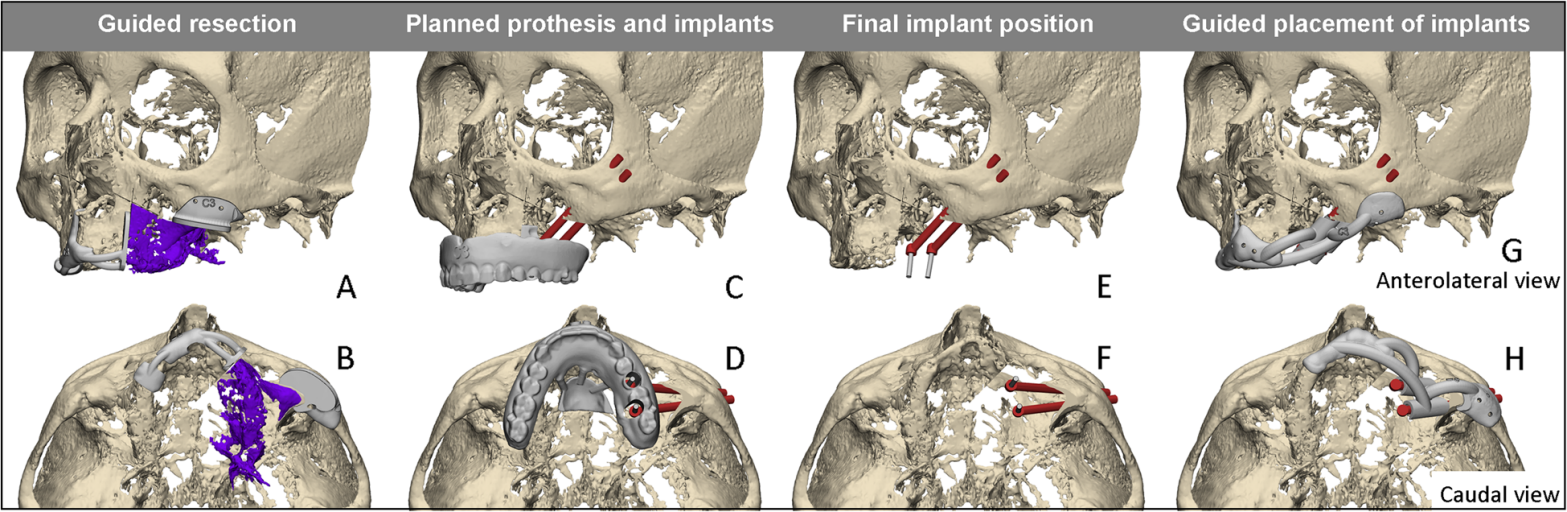
Overview of 3D VSP workflow, including the anterolateral view (upper row) and the matching caudal view (lower row). The 3D VSP starts with planning of the maxillectomy including the design of the cutting guide, with which the aim is to remove the purple part representing tumor removal (**a**, **b**). Hereafter the obturator prosthesis position is planned in the defect with the pre-planned screw access holes (**c**, **d**). The positions of the zygomatic implants are planned backward from this optimal position of the obturator prosthesis (**e**, **f**). The final step includes the design of the drilling and placement guide of the zygomatic implants (**g**, **h**)

To mimic the clinical problem of a maxillary defect, typical examples of maxillary tumor resection surgeries were planned virtually (Fig. 1a, b). 3D VSPs are created that included partial resection of the maxilla, leaving a maxillary defect, based around an assumed tumor volume that would be suitable for obturator prostheses supported by zygoma implants. The defects created in this experiment were classified as low-level Brown Class 2b maxillectomies [13].

Based on the 3D VSP, surgical cutting guides were designed and printed to transfer the resection plan to the cadavers. Next, an obturator prosthesis was designed in the software matching the virtually created defect. Pre-existent dentures were not available for any of the cadavers. The maxillary soft tissues were segmented in order to design digital maxillary dentures as base templates for the final obturator prostheses. Implementing the digital obturator prostheses completed the VSP and enabled the digital planning of the prosthetic implant platform positions.

The position of the zygomatic implants was planned backward from the position of the prostheses. The zygomatic implant heads, to support and fixate the prostheses to the zygomatic implants, were placed in the most ideal prosthodontic positions, slightly palatal from the occlusional plane (Fig. 1c, d). In a vertical dimension, enough space for a future bar superstructure and acrylic was taken into account (Fig. 1e). Horizontally, the spacing between the prosthetic implant platforms was carefully chosen in order to fit a clip retention system and to enhance any necessary cleaning of the implants (Fig. 1f).

The positioning of the zygomatic implant was planned with the tip of the implant placed in the lateral cortical bone of the zygomatic complex. The assumption was that placing the apical part of the implant in the cortical bone provides optimal primary stability and will cover the bone on the lateral side of the implant.

The preferred apical and abutment positions of the zygomatic implants, implant lengths, and obturator prosthesis were designed virtually. Subsequently, patient specific implant drill and placement guides were designed based on the final virtual set-ups (3Matic Medical, Materialise, Leuven, Belgium) (Fig. 1g, h). The drilling/placement guides were developed to fit the following bone structures: alveolar ridge, nasal floor, and zygomatic arch. The guides were printed from polyamide, produced according to the ISO 13485 standards for medical devices, at Oceanz (Ede, the Netherlands). The study ultimately resulted in an advanced implant guide design. The addition of centered channels in the drill-guide enables angled cuts and the length of the channels form an integral depth stop for the drill. The insertion of stainless steel (316 L) milled drill sleeves in the channels should minimize deviation of the drill trajectories. The maxillary bone-supported part included an extension to the nasal aperture to verify good positioning of the guide [14] and was connected with crosslink arms to the zygomatic bone-supported part. In addition, the guide was supplied with holes for temporary fixation with mini screws.

### Surgical procedure

The cadaver surgery was split into two series to evaluate the findings and, if necessary, to adjust the guides and/or obturator prosthesis before the second test. The surgery was performed by OMF surgeons involved in the planning process, and the supportive visual documentation of the planned guide position was always present in the operating room. Two cadaver heads were thawed before surgery for the first session, and the other three were thawed later for the next session.

**Fig. 2:**
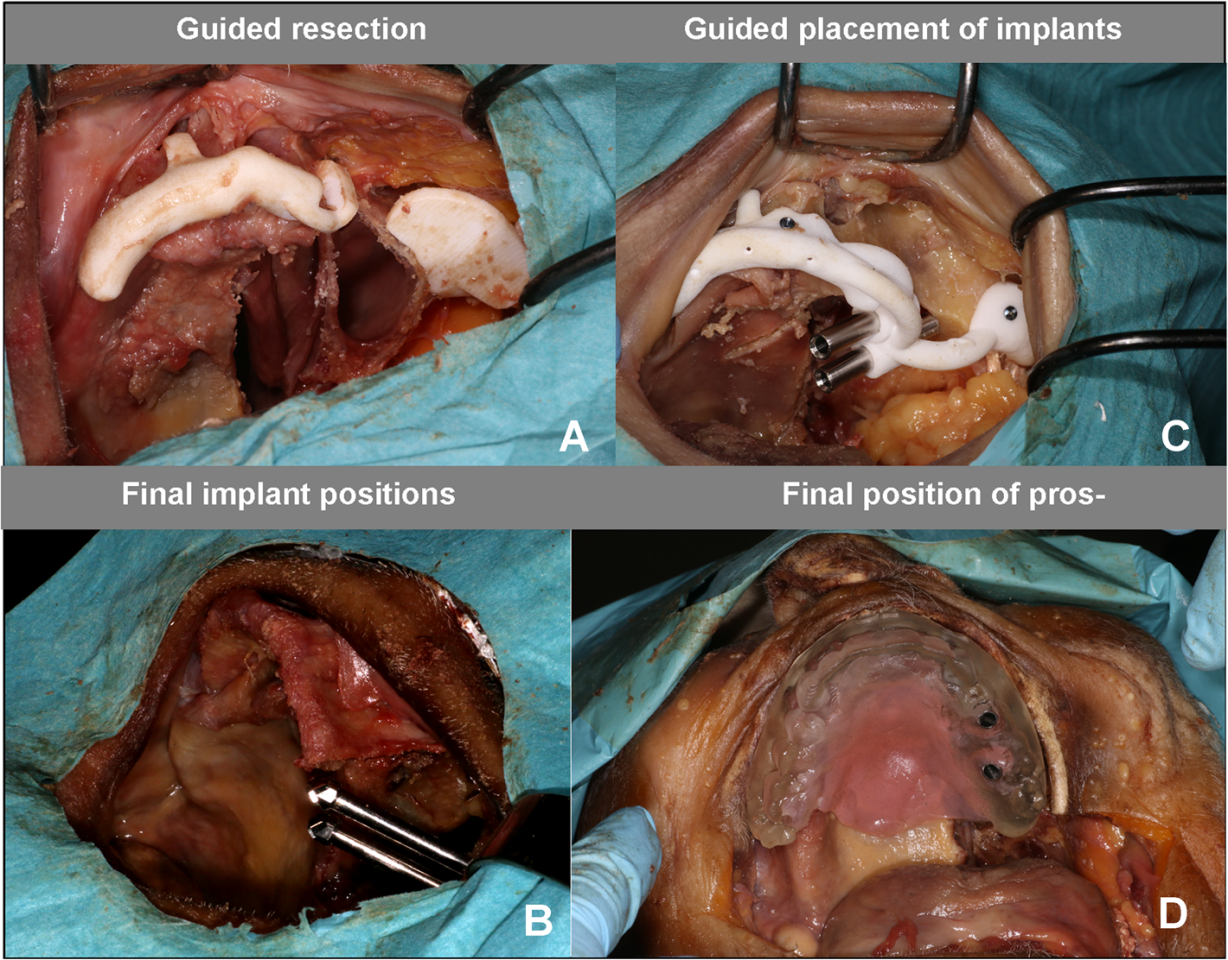
Overview of the surgical procedure. The working method starts with left-sided maxillectomy guided by the surgical template (**a**). Next, the drill-guide was fixed to the bone on two anatomical locations (zygoma and premaxilla) using 2.0 mm cortical locking screws and two zygomatic implants were guided placed in the planned positions (**b**). Finally, prosthetic cylinders were fixated to the obturator prosthesis with light cured resin to fixate the prosthesis firmly on the zygomatic implant abutments (**d**)

To create the Class IIb maxillary defects, the cutting guides were placed on the denuded bone of the maxilla and the zygoma (Fig. 2a). The stability and fit of the bone-supported cutting guide was verified. The left-sided maxillectomies were guided by the surgical templates. The resected specimen, mimicking a tumor resection, was removed, resulting in a Class IIb defect. The cutting guides were removed and subsequently the implant drilling guide was fitted and placed.

**Fig. 3:**
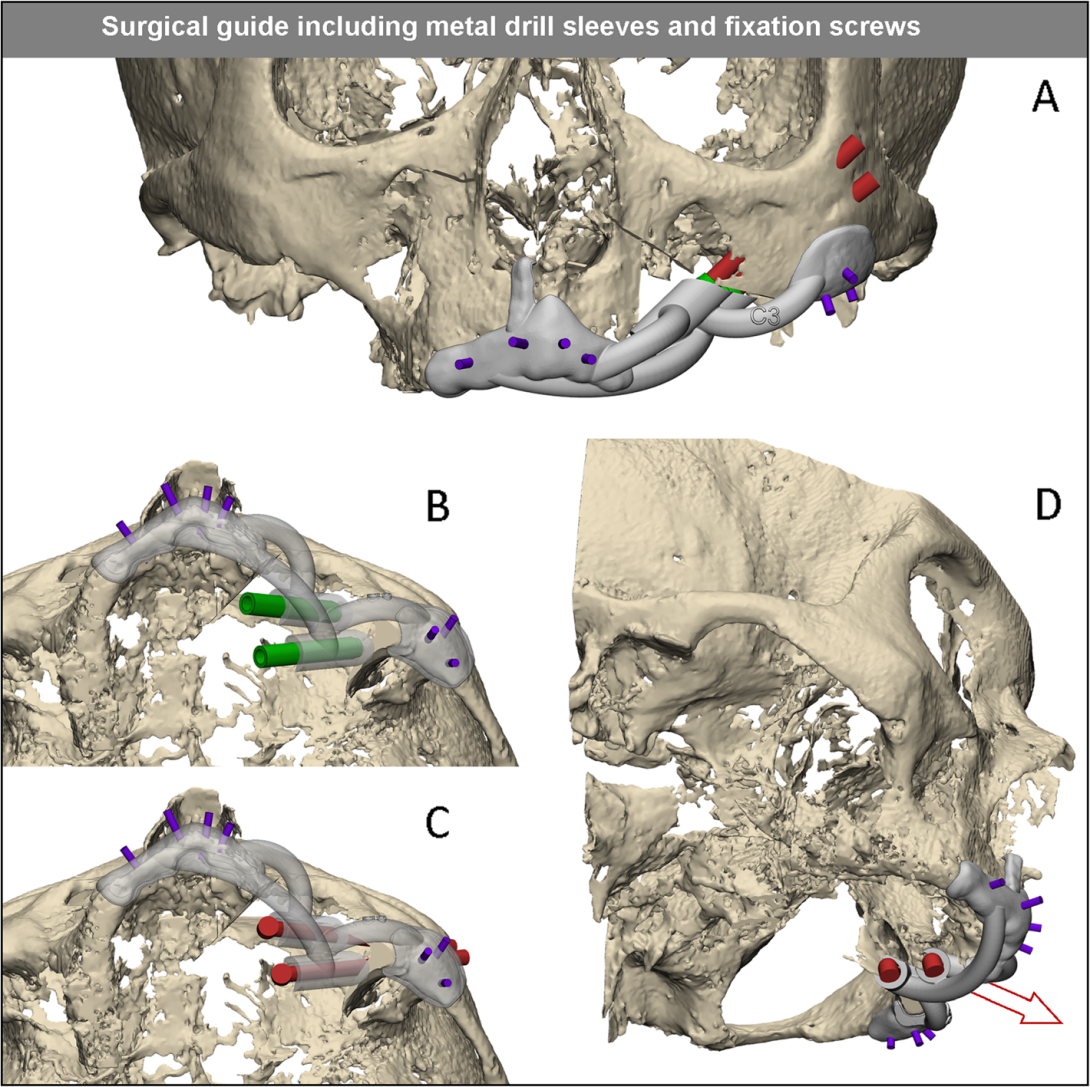
Detailed view of the guide design. In gray, the body of the 3D printed polyamide guide (**a**). In red, the planned zygomatic implants. In green, the metal inserts used during drilling (**b**). The metal insert is pushed in the guide by the surgeon during surgery. After drilling the trajectory, the metal insert is removed to accommodate the thicker diameter of the implant. The direction as well as the depth of the implant is set by the design and physical dimensions of the guide (**c**). After insertion of the implants, the guide is removed by first removing the mini screws (purple) and then removing the guide in the opposite direction of the slots in the implant guide (red arrow) (**d**)

The precise alignment to the underlying bone structures was verified. The drill-guide was fixed to the bone on two anatomical locations (zygoma and premaxilla) using 2.0 mm cortical locking screws (KLS Martin, Tuttlingen, Germany) (Figs. 2c and 3a). After drilling, the implant beds through the guide, according to the drill sequence for oncological zygomatic implants (Southern implants, South Africa), the metal sleeves were removed (Fig. 3b). The guide was designed to direct the angle and depth of the implant placement (Fig. 3c). The VSP planned implant lengths were placed and the final prosthetic platform position was checked by the maxillofacial prosthodontist and the guide was removed (Figs. 2b and 3d). The obturator prosthesis was then fitted, which provided the surgical team with a visual check as to whether the emergence of the zygomatic prosthetic platforms was favorable or not, in relation to the pre-planned slots in the obturator prosthesis. The surgical procedure was finalized by fixating the obturator prosthesis. Non-engaging prosthetic cylinders (Southern implants, South Africa) were fixed to the obturator prosthesis with light cured resin to fix the prosthesis firmly on the zygomatic implant abutments (Fig. 2d). The obturator prosthesis was checked for balance support on the contralateral side of the residual maxilla. After the surgical procedure, the obturator prostheses were removed and the heads underwent a post-operative cone beam CT scan to analyze implant accuracy.

In preparation for the second cadaver operation session, two alterations were made to the working method. The first alteration was the use of more rigid and solid crosslink arms on the drilling guide, to minimize guide movement due to vibrations during drilling. Secondly, longer mini screws were used to fixate the guides to the bone. The longer 8 mm screws were better for retention in the slightly porous cadaver bone.

### Outcome measures

The primary outcome of this study was the fit of the prosthetic cylinders connected to the placed zygomatic implants in the preoperative positioned slots of the obturator prostheses. It was noted if the obturator prostheses needed adjusting to fit the cylinders. Both surgical and prosthetic steps were based on one virtual surgical plan and had to tally with the final positions of the prosthetic implant platform above the designed screw access holes in the dental arch of the obturator prosthesis. In all five cadavers, the support for the obturator prostheses had to be on the remaining maxilla and should match the surgical resection. A placement accuracy of within 3 mm of the prosthetic cylinders in the slots were considered to be successful for a prosthesis, resulting in a passive fit.

**Fig. 4:**
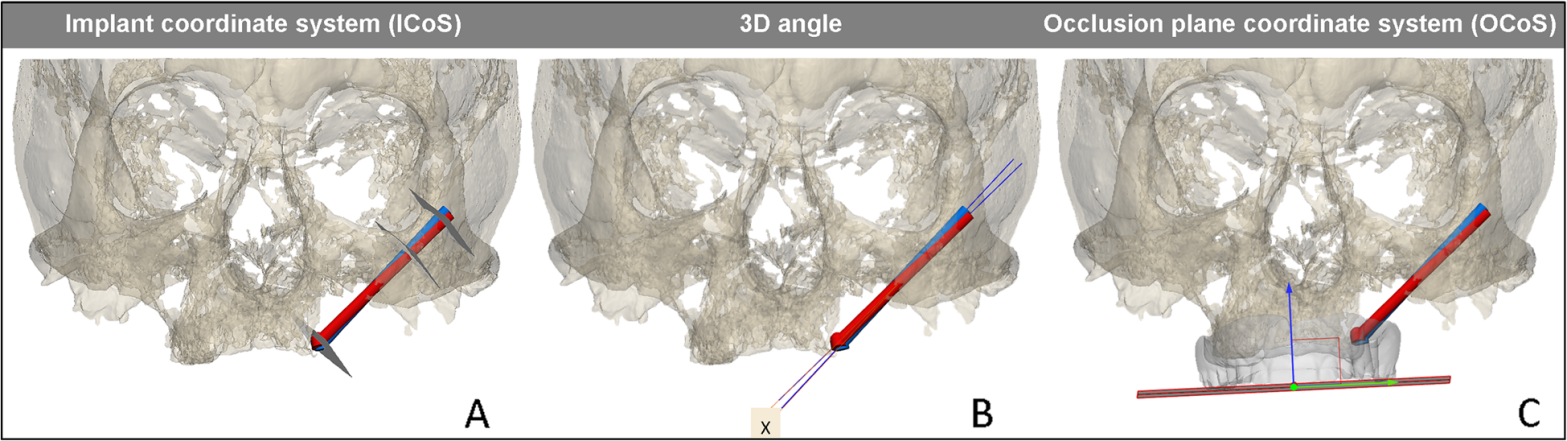
Overview of the several types of measurements and reference planes or coordinate systems for assessing the accuracy of zygomatic implant placement derived from post-op CBCT. In red, the planned zygomatic implant position; in blue, the postoperative zygomatic implant position. Left: the implant coordinate system (ICoS) including the three reproducible reference planes in which the accuracy is measured; the center of the zygomatic implant head, bone entry point of the implant, and bone exit point of the implant. Middle: 3D angular deviation between 3D planned position and post-operative implant position. Right: occlusion plane coordinate system (OCoS). A plane parallel to the prosthetic occlusional plane is defined; perpendicular to this plane is the blue arrow. This arrow indicates the direction in which the abutment height accuracy is calculated

A secondary outcome measure for 3D planned series was the zygomatic implant placement accuracy. The post-operative CBCT-data were obtained in a similar fashion as for the pre-operative CBCT. The post-operative maxillae were segmented and, the implant positions were matched with the 3D VSP. The post-operative implant positions are determined by two observers. The most distal part of the long axis of the implant was used as the abutment position (Fig. 4a), so that the results were not dependent on a rotation along the long axis. The entry and exit positions in the zygomatic bone were defined by the intersection of this long axis with the virtual maxilla.

Two coordinate systems were defined:

1. The Implant’s Coordinate System (ICoS); the *z*-axis runs along the long axis of each planned implant.
2. The Occlusion Coordinate System (OCoS); congruent with the axial, saggital, and coronal planes, where the axial plane is defined by the occlusion plane of the virtual obturator prostheses.

The planes perpendicular to the *z*-axis, and running through the planning’s abutment point, defined the entry and exit points for the ICoS measurements. The intersections of the implant long-axes with these planes were defined as the corresponding points of the outcome. Then, the distance between the abutment, the entry and exit points, and their corresponding points were defined. Also, the 3D angular deviations between the planning’s and the outcome’s long axes were determined (Fig. 4a–c). Unpaired *t* tests were conducted as well as the intraclass correlation (ICC) of the implant reconstruction between the two observers.

## Results

A total of 10 zygomatic implants were placed in 5 cadaver heads. Some minor alterations were made to the guides between the first and second session. Rigidity was improved by increasing the diameter of the crosslink arms and a window was added to the guide to give the surgeon a direct view of the entry point of the zygoma bone. This enabled minimal movement of the guides due to vibration during drilling. Also, longer cortical osteosynthesis screws were used in the second series for guide fixation to the bone. The longer 8 mm screws allowed better retention in the slightly porous bone. Other than this, the planning and guide design was not essentially changed.

### Outcome measure 1: position of the prosthetic implant platform in relation to the obturator screw access hole

Non-engaging prosthetic cylinders were screwed in place and the alignment with the screw access holes was checked. All five obturator prostheses could be placed with high accuracy. The outcome in the horizontal as well as in the vertical dimension was within the 3 mm leeway space for the prostheses. The fit of the preplanned obturator prosthesis was adequate and well balanced on the remaining maxillary structures in all cases. The prosthetic cylinders were integrated into the obturator prostheses successfully in all cases, without needing any further prosthetic adjustments.

### Outcome measure 2: implant placement accuracy

With the aid of the VSP, the drill and placement guides, a total of 10 zygomatic implants were placed. The implant lengths varied between 45 mm and 55 mm and where placed with a mean entry point deviation of 1.20 ± 0.61 mm and a 3D angle deviation of 2.97 ± 1.43° (range 1.0–5.5°). The 3D accuracy of the abutment positions was 1.19 ± 1.31 mm. The accuracy of the abutment position in the occlusional plane was 1.77 ± 1.31 mm, with a height accuracy of 1.03 ± 0.85 mm. The complete accuracy results can be seen in Tables 1 and 2). The intraclass correlations (ICC) between the first and second observer for the positions of the abutment, entry-point, exit-point, and 3D angle were 0.91 mm, 1.00 mm, 0.97 mm, and 0.98°, respectively.

**Table 1:** Accuracy data. Result of the post-op analysis of the implant coordinate system (ICoS) measurements

ICoS measurements Cadavers	Mean (±SD)	Min	Max
Abutment (mm)	1.19 (**±** 0.63)	0.1	2.1
Entry-point (mm)	1.20 (**±** 0.61)	0.4	2.1
Exit-point (mm)	2.12 (**±** 1.24)	0.7	4.1

**Table 2:** Accuracy data. Result of the post-op analysis. Descriptive statistics of the occlusion coordinate system (OCoS) measurements

OCoS deviations Cadavers	Mean (±SD)	Min	Max
Abutment in occlusal plane (mm)	1.77 (**±** 1.31)	0.8	5.3
Abutment height from occlusal plane (mm)	1.03 (**±** 0.85)	0.1	3.2
Axial angle (°)	2.07 (**±** 2.63)	0.8	5.2
Coronal angle (°)	0.99 (**±** 2.32)	0.7	4.2
Sagittal angle (°)	1.48 (**±** 3.59)	0.9	7.5
3D angle (°)	2.97 (**±** 1.43)	1.0	5.5

No statistical significant differences were found between the mean values of the ventral and dorsal implants (*P* > 0.05). No statistical differences were found between implants placed in the first session and in the second session.

## Discussion

This study shows that it is possible to accurately apply 3D virtual planning to guided surgery and implant-retained maxillary prosthetic rehabilitation in one procedure. The described treatment protocol merges 3D virtual surgical planning and 3D virtual prosthetic planning into a single overall treatment modality. Full 3D prosthetic planning offers insight into a maxillary defect size, ahead of ablative surgery, and enables highly accurate prosthetic-driven implant planning as well. Primary implant placement at the time of ablative surgery has been shown to be an effective means of accelerating rehabilitation, along with early loading protocols [5, 7, 15]. Placement of implants in one procedure with ablative surgery is an advantage, especially when the oncology treatment requires post-operative radiotherapy for disease control because it avoids the issue of secondary surgery in irradiated tissues [5, 7, 10, 16]. The use of virtual planning techniques to enable accurate guided placement of endosseous implants is now a common procedure, and 3D-assisted planning to determine the ideal zygomatic implant position is used regularly. Some case reports mention surgical navigation as a viable technique to transfer planned implant positions [11, 17, 18]. However, zygomatic implant placement is challenging because of the long drill path and complex anatomic components. The main drawback of current visualization techniques is the difficulty of maintaining the drilling handpiece steady in the right direction, and transferring the surgical view from the navigation display to the operative site [17, 18]. A more reliable transferring method for planned zygomatic implant positions seems to be 3D printed drilling/placement templates. This study demonstrates that it is possible to design one zygomatic drilling template to provide an accurate means of translating the virtual 3D plan. It has been reported that immediate loading of zygomatic implants is a viable treatment option. Boyes-Varley et al. [10] described a workflow where the prosthesis was placed immediately after implant placement in close contact with the implants, but not screw-fixed on the prosthetic implant heads. The prosthesis was resting on top of the zygomatic implants and the obturator prosthesis was fixated to the palatal bone with cortical-osteosynthesis screws. In this study, the obturator prosthesis could be fixated rigidly to the implants without the need for any other fixation, due to the accurate 3D planning of the screw holes in the obturator.

The use of an implant-retained surgical obturator may have a positive effect on the primary stability of the zygomatic implants during bone healing. The obturator prostheses used in this study functioned as an external rigid fixation device that splints the implants together. Primary implant stability is provided just with the zygomatic anchorage, while the coronal fixation is provided by the implant fixated obturator prosthesis. Although the obtained stable prosthetic situation is believed to be effective for some months, eventually it is recommended to pursue cross arch splinting of the zygomatic implants in final prosthetics to contribute to implant survival.

It is reasonable to assume that knowledge of the planned resection automatically provides 3D visualization of the necessary obturator outline to restore oral function. In this study, a treatment protocol is described for immediate prosthetic rehabilitation with immediate loading of the zygomatic implants. Restoring oral function immediately after ablative surgery, in one procedure with implant placement, obviates the need for fitting, placing, and adapting the prostheses. After maxillectomy, the frequent necessity of adjuvant radiotherapy limits the possibility of achieving sufficient retention for a conventional obturator prosthesis. An implant-retained obturator prosthesis allows for repeated removal to check the oncological defect visually, or in the event of complications. The addition of subsequently placing a fixed-removable obturator prosthesis during surgery is a major step to shortening the time of prosthetic delivery and implant utilization. It can be anticipated that the number of prosthetic interventions post-operatively will be less compared to conventional prosthetic planning in which retention is more difficult to obtain. We assume that such patients can recover earlier and better before the often necessary radiotherapy starts and the hospital visits for prosthetic aftercare will be minimized in early post-operative phase.

## Conclusions

This report has introduced a full 3D virtual workflow to enable immediate implant retained prosthetic rehabilitation after a maxillectomy. Zygomatic implants should be placed very accurately in the planned positions using the novel designed patient specific drilling and placement guides, allowing screw-retained fixation of an obturator prosthesis. This concept will be verified next in patients with maxillary cancer who have been planned for prosthetic rehabilitation with an obturator prosthesis.

## Data Availability

The datasets used and/or analyzed during the current study are available from the corresponding author on reasonable request.

## Abbreviations

3D: Three dimensional
VSP: Virtual surgical plan
ICoS: Implant Coordinate System
OCoS: Occlusion Coordinate System
ICC: Intraclass correlation
